# Intrathecal B cell-related markers for an optimized biological investigation of multiple sclerosis patients

**DOI:** 10.1038/s41598-022-19811-3

**Published:** 2022-09-30

**Authors:** Sawsan Feki, Mariem Damak, Salma Sakka, Yesmine Ben Ali, Sabrina Mejdoub, Nadia Bouattour, Hend Hachicha, Chokri Mhiri, Hatem Masmoudi

**Affiliations:** 1 Laboratory of Immunology, Habib Bourguiba University Hospital, Faculty of Medicine, University of Sfax, Sfax, Tunisia; 2 Department of Neurology, Habib Bourguiba University Hospital, Clinical Investigation Center, Faculty of Medicine, University of Sfax, Sfax, Tunisia

**Keywords:** Biological techniques, Immunology, Neuroscience

## Abstract

In multiple sclerosis (MS) disease, the importance of the intrathecal B cell response classically revealed as IgG oligoclonal bands (OCB) in cerebrospinal fluid (CSF) was reaffirmed again in the recently revised diagnostic criteria. We aimed to optimize Laboratory investigation by testing the performance of new B cell-related molecules in CSF (Ig free light chains (FLCκ and λ) and CXCL13 (*B-Cell Attracting chemokine1*)) for MS diagnosis. 320 paired (CSF-serum) samples were collected from 160 patients with MS (n = 82) and non-MS diseases (n = 78). All patients benefited from IgG index determination, OCB detection, CSF CXCL13 and FLC (κ and λ) measurement in CSF and serum for metrics calculation (κ/λ ratio, FLC-related indexes, and κFLC-intrathecal fraction (IF)). CXCL13 and FLC metrics in CSF were higher in patients with MS and positive OCB. As expected, κFLC metrics—in particular, κFLC index and κFLC IF—had the highest accuracy for MS diagnosis. κ index showed the best performance (sensitivity 83% and specificity 91.7%) at a cut-off of 14.9. Most of the FLC-related parameters were positively correlated with IgG index and the level of CXCL13. In conclusion, the quantitative, standardizable, and technically simple CSF FLCκ metrics seem to be reliable for MS diagnosis, but could not replace OCB detection. CXCL13 appears to be an effective parameter reflecting the intrathecal B cell response. An optimized way for CSF testing combining the conventional and the new B cell-related parameters is proposed in this study.

## Introduction

The etiology of multiple sclerosis (MS), a common neuroinflammatory and neurodegenerative disorder of the central nervous system (CNS)^1^, which affects predominantly young adults and leads to neurological disability^2,3^, is still unknown. An auto-reactive cellular and humoral immune response responsible for multifocal myelin destruction throughout the CNS seems to be the major pathological process. Although MS is traditionally considered a predominantly T cell-mediated autoimmune disease, several lines of evidence have supported the idea that B cell may be a major contributor to the disease pathogenesis^4,5^. In particular, studies of the cerebrospinal fluid (CSF) have provided important highlights into the B cell response in MS. The first evidence was identified decades ago with the identification of unique immunoglobulin (Ig) G fractions in the CSF^6^ produced by intrathecal clonal B cell populations^7^ and revealed as oligoclonal bands (OCB) in up to 90% of all patients with MS^8^. This biological sign is considered as a diagnostic hallmark of the disease. Other findings have supported the role of B cells and intrathecal Ig in the pathogenesis of MS, which includes (i) detection of B-cell infiltrates in the CNS (B cell-predominant lymphoid aggregations called germinal center (GC)-like structures), in particular within lesions (especially active lesions of patients with relapsing–remitting disease)^9,10^, (ii) elevated levels of antigen-experienced, class-switched memory B cells and antibody-producing plasma cells in the CSF (relative to their levels in the blood) of patients with MS (iii) Ig-related genes upregulation in cortical sections of patients with MS^11^, and (iv) efficacy of B cell-targeting therapies (e.g., rituximab, ocrelizumab)^12,13^.

Furthermore, much evidence suggests that the inflamed CNS provides different factors supporting the long-term survival of B-lineage cells in the environment—such as TNF ligand superfamily member 13B, also known as BAFF or BLyS and chemoattractants—such as stromal cell-derived factor 1 (also known as C-X-C motif chemokine ligand 12 (CXCL12)) and CXCL13^14–16^. It has been particularly reported that CSF levels of CXCL13 correlate with the clinical response to B cell-targeting therapies^17^.

An early and accurate MS diagnosis is crucial for the clinical management of patients since recent therapeutic alternatives (disease-modifying therapies) effectiveness depends on the stage of disease diagnosis^18,19^. According to the last 2017 revisions of the McDonald MS diagnostic criteria, the diagnosis is based on clinical symptoms, MRI imaging, and biological investigation, including cerebrospinal fluid (CSF) testing^20^*.*

From the Laboratory’s point of view, the detection of the IgG oligoclonal bands (OCB) in CSF, which has been reported since the 1970s^21,22^, has gained more importance in these recently revised MS diagnostic criteria. This biological sign reflecting an intrathecal synthesis (IS) of IgG antibodies and considered an immunological hallmark of MS, is classically revealed using the gold-standard technique, which is the CSF Isofocusing test. Other quantitative tests (total IgG index calculation and/or the Reiber diagram) are also useful in this context. However, in routine practice, it is not rare to be faced with some confusing situations (OCB-positive alternative diagnosis, OCB-negative MS…) and some technical issues related mainly to the standardization of OCB detection.

Recently, standard assays to measure other B cell-related biomarkers levels in CSF (the two free light chains (FLC) kappa (κ) and Lambda (λ) and the chemokine CXCL13 responsible for B cell recruitment within the CNS) have been studied, and seem to improve the detection of intrathecal B cells activity^23–29^. These B cell-related parameters have been reported to be increased in the CSF of MS patients and were proposed as quantitative standardizable diagnostic and/or prognostic biomarkers for MS. In particular, κ-FLC level in CSF seems to be a more sensitive marker of IS of IgG compared with OCBs^25,30^.

We aimed to determine the additional value of testing the new B cell-related markers in CSF (FLC metrics and CXCL13) for the detection of the intrathecal humoral response and MS diagnosis in order to optimize the Laboratory investigation in this context.

## Materials and methods

### Subjects

During the 4 years study period (January 2018–December 2021), patients from the Department of Neurology of the Habib Bourguiba Hospital (Sfax, Tunisia) who are suspected of having an inflammatory disorder of the CNS were included. Lumbar puncture was performed for a diagnostic purpose (no disease-modifying drugs or glucocorticosteroids at the time of lumbar puncture). Paired CSF and serum samples were collected from patients and addressed to the Laboratory of Immunology of Habib Bourguiba Hospital for Immunological investigation (IgG index calculation, Reiber Diagram, and CSF Isofocusing test). Patients’ clinical and radiological records have been retrospectively consulted. The MacDonald 2017 revised criteria of MS were used for the disease diagnosis. This diagnosis was made within 2 weeks of the Lumbar puncture for all of the patients. The degree of neurological impairment in patients with MS was evaluated using the expanded disability status scale (EDSS) at the time of sampling.

Our study population consisted of 160 patients with neurological disorders (Table 1), which was divided into 2 groups: an MS group (n = 82) and a non-MS group used as a control group (n = 78). Patients benefited from FLC measurement in CSF and serum for respective metrics and ratio calculation, in addition to the routine immunological CSF investigation (Table 1). The level of CXCL13 chemokine was also determined in the CSF of all patients.

**Table 1:** Clinical, radiological and immunological features of the study population.

Total n = 160	MS group n = 82	Non-MS group n = 78	*p-*value
Gender	22 M/60 F	34 M/44 F	0.08
Age (years) [median (range)]	36 (18–67)	40.5 (20–67)	0.2
Diagnosis	MS	11 Neuro-Behçet’s	–
		10 NMOSD	
		9 Neuro-sarcoidosis	
		7 Neuro-Sjogren’s Syndrome	
		5 Neuro-lupus	
		7 Vasculitis	
		6 Meningoencephalitis	
		5 Guillain Barre syndrome	
		18 Other diagnoses (neurological malignancies …)	
**Clinical form**			
Relapsing–remitting	72	–	
Progressive primary	3	–	–
Progressive secondary	7	–	
Optical neuritis	35	19	0.07
**MRI**			
Cerebral abnormalities	78	33	–
Myelitis	34	21	
EDSS mean (range, SD)	2.43 (0–5; ± 2.1)	–	–
**Total IgG index**			
Mean (range; ± SD)	1.05 (0.47–3.38; ± 0.62)	0.74 (0.37–2.55; ± 0.4)	< 0.001
< 0.7	26 (32%)	58 (74.3%)	
≥ 0.7	56 (68%)	20 (25.7%)	
**Reiber diagram**			
Blood–brain barrier dysfunction	10 (12.2%)	26 (33.3%)	0.002
Intrathecal synthesis of IgG	49 (59.8%)	20 (25.6%)	< 0.001
**CSF isofocusing**			
OCB (+)	63 (76.8%)	27 (34.6%)	< 0.001
OCB (−)	19 (24.2%)	51 (65.4%)	

*MS* multiple sclerosis, *NMOSD* neuromyelitis optica spectrum disorder, *MRI* magnetic resonance imaging, *EDSS* expanded disability status scale, *CSF* cerebrospinal fluid, *OCB* oligoclonal band.

In this study, all methods were carried out in accordance with relevant guidelines and regulations. In addition, the study experimental protocols were approved by the Regional Committee for the protection of persons (Ministry of Health) (CPP SUD No 0407/2022).

Written informed consent was obtained from all subjects and/or their legal guardian(s).

### Methods

In our routine practice, we receive couples of samples (CSF specimen from a lumbar puncture and venous blood sample) for immunological investigation of patients with suspicion of inflammatory disorders of the CNS. The collected specimens are centrifuged, aliquoted, and frozen at − 80 °C. Each patient’s serum and CSF samples were analyzed in parallel, at the time of diagnosis using a CSF Isofocusing test which includes 2 steps according to the manufacturer’s instructions (Hydragel 3 CSF Isofocusing; Hydrasys; Sebia^®^, France): an isoelectric focusing on agarose gel and an immunofixation with peroxidase-labeled anti-IgG antiserum. The Isofocusing test profile allows the comparison of IgG distribution in both fluids (IgG OCB detection). A positive pattern (OCB+) is defined by the presence of at least two additional OCB in CSF in comparison with serum. Measurement of total IgG and albumin levels in sera and CSF for the calculation of *Tibbling et Link* IgG index using the formula: [(IgG CSF/IgG serum)/(Albumin CSF/Albumin serum)] was performed using the automatic nephelometric method (BN Prospect, Siemens^®^, Germany). These 2 routine tests are used in such context to detect an IS of IgG (revealed as additional OCB (n ≥ 2) in CSF in comparison with serum and/or a value of IgG index > 0.7). We used Reiber Diagram^31^ to check the blood–brain barrier status.

The concentrations of κFLC and λFLC in CSF and serum were measured according to the same nephelometric method. In the case of undetectable FLC concentrations, the corresponding detection limit (CSF κFLC, 0.174 mg/L, CSF λFLC, 0.47 mg/L) was used as the level of the parameter. Regarding the CXCL13 level in CSF, it was determined using an immunoenzymatic technique (ELISA) (Euroimmun^®^, Germany). The lower detection limit for the CXCL13 ELISA is 4.0 pg/mL.

To analyze the different FLC metrics, different formulas were used. The κFLC index and λFLC index were calculated according to the following formulas:

- [CSF κFLC (mg/L)/serum κFLC (mg L)]/[CSF albumin (mg/L)/serum albumin (mg/L)] and [CSF λFLC (mg/L)/serum λFLC (mg/L)]/[CSF albumin (mg/L)/serum albumin (mg/L)], respectively.

We also calculated the FLC ratio in CSF (CSF κFLC/CSF λFLC), the κIgG index [(CSF κFLC (mg/L)/serum κFLC (mg/L)]/[CSF IgG (mg L)/serum IgG (g L) × 100)] and the λIgG index [(CSF λFLC (mg L)/serum λFLC (mg L)/CSF IgG (mg L)/serum IgG (g L) × 100)].

We evaluated the sensitivity of a test as [true positives/(true positives + false negatives)] and the specificity as [true negatives/(true negatives + false positives)]. The positive predictive value (PPV) was calculated as [true positives/(true positives + false positives)] and the negative predictive value (NPV) as [true negatives/(true negatives + false negatives)].The Youden index (or Youd’s J statistic) is defined as J = sensitivity + specificity − 1.

In addition, the software “FLC-κ statistics and graphic program” (Albaum W. CSF-App; CSF Research Tool/Reibergrams; FLC-K Statistics and Graphic program; 2020 [access: 12/04/2020]. http://www.albaum.it) for FLCκ calculates the κloc (mean) and κFLC inthrathecal fraction IF (mean) values for the data taken from Excel tables. The percentage FLC IF is calculated by comparing QFLC to a previously empirically determined, albumin quotient-dependent reference limit. A graphical result could also be generated for FLCκ using the software.

### Statistical analysis

Statistical analysis was performed using SPSS 20.0 software. To analyze quantitative variables, we calculated means and SEM (standard error of the mean) and performed a comparison using Student-T test. In case of a skewed distribution from the normality, medians (1st Quartile–3rd Quartile) were calculated and compared using the Mann–Whitney U test. Correlations were studied using the Spearman test. A *p-*value < 0.05 was defined as statistically significant. The κ index graph was created using the free software “Albaum” (www.albaum.it). Roc curves and Box plots were generated using SPSS software.

## Results

In this study, in addition to the conventional tests (IgG index calculation, Reiber Diagram, CSF Isofocusing test) (Table 1), we investigated other B lymphocyte-related parameters in CSF (the κFLC and λFLC metrics, the chemokine CXCL13) and studied their performance for MS diagnosis and their correlation with the intrathecal humoral immune response (Table 2).

**Table 2:** Quantitative results of the different B cell-related parameters in CSF in the study population.

	MS n = 82	Non-MS n = 78	*p* value*	OCB positive patients n = 90	OCB negative patients n = 70	*p* value*
Qalb (CSF/serum albumin) mean (SEM)	4.7 (0.3)	6.8 (0.6)	**0.002**	4.7 (0.40)	6.6 (0.53)	** < 0.001**
QIgG (CSF/serum IgG) mean (SEM)	4.6 (0.32)	5.7 (0.9)	0.2	5.5 (0.70)	4.3 (0.68)	0.23
Ig G index mean (SEM)	1.05 (0.67)	0.72 (0.04)	** < 0.001**	1.15 (0.06)	0.58 (0.02)	** < 0.01**
Intrathecal synthesis of IgG according to Reiber (%)	72.7	39	** < 0.001**	87.9%	12%	** < 0.001**
Serum κFLC (mg/L) mean (SEM)	16.15 (0.75)	18.75 (1.3)	0.08	16.4 (0.71)	18.1 (0.98)	0.15
CSF κFLC (mg/L) mean (SEM)	4.3 (0.56)	0.92 (0.16)	** < 0.001**	4.6 (0.57)	0.67 (0.14)	** < 0.001**
Serum λ FLC (mg/L) mean (SEM)	20.3 (1.1)	25.1 (1.77)	**0.025**	21.4 (1.05)	22.4 (1.57)	0.6
CSF λ FLC (mg/mL) mean (SEM)	1.1 (0.17)	1.1 (0.26)	0.9	1.3 (0.23)	0.68 (0.10)	**0.012**
κ/λ ratio mean (SEM)	8.2 (1.4)	2.1 (0.66)	**0.003**	9 (1.4)	1.3 (0.29)	** < 0.001**
κ index mean (SEM)	77.9 (12)	10.9 (2.6)	** < 0.001**	79.4 (11.9)	10.3 (4.61)	** < 0.001**
λ index mean (SEM)	17.7 (4.67)	7.6 (1.40)	**0.043**	14.7 (1.73)	11.6 (6.84)	0.65
κIgG index mean (SEM)	5.95 (0.56)	1.52 (0.23)	** < 0.001**	4.9 (0.5)	2.9 (0.64)	**0.01**
λIgG index mean (SEM)	1.5 (0.25)	0.94 (0.12)	0.07	1.3 (0.2)	1.1 (0.17)	0.5
κFLC IF (%)	86.87	35.1	** < 0.001**	86.74	39.87	** < 0.001**

*FLC* free light chain, *MS* multiple sclerosis, *OCB* oligoclonal bands, *CSF* cerebrospinal fluid, *FLC IF* free light chain intrathecal fraction, *SEM* standard error of the mean.

*Student-t test.

Significant *p* values are in bold.

### Free light chain-related parameters in CSF

#### FLC levels and ratio in CSF

In this study, the mean value of κFLC (but not λFLC) level in CSF was significantly higher in the MS group than in the control group. When comparing all patients according to the OCB positivity, both types of FLC were significantly higher in the OCB-positive group than in the OCB-negative group.

We also noticed that κFLC and λFLC were undetectable in CSF of 26/160 (16.25%) and 62/160 (38.75%) included patients, respectively. Among the 26 patients with an undetectable level of κFLC in CSF, only 4 were MS patients, and all of them were OCB negative. It is interesting to note that for the remaining 22 non-MS patients with an undetectable level of κFLC, 19 were OCB-negative, 2 OCB-positive, and 1 with one band in CSF.

**Figure 1:**
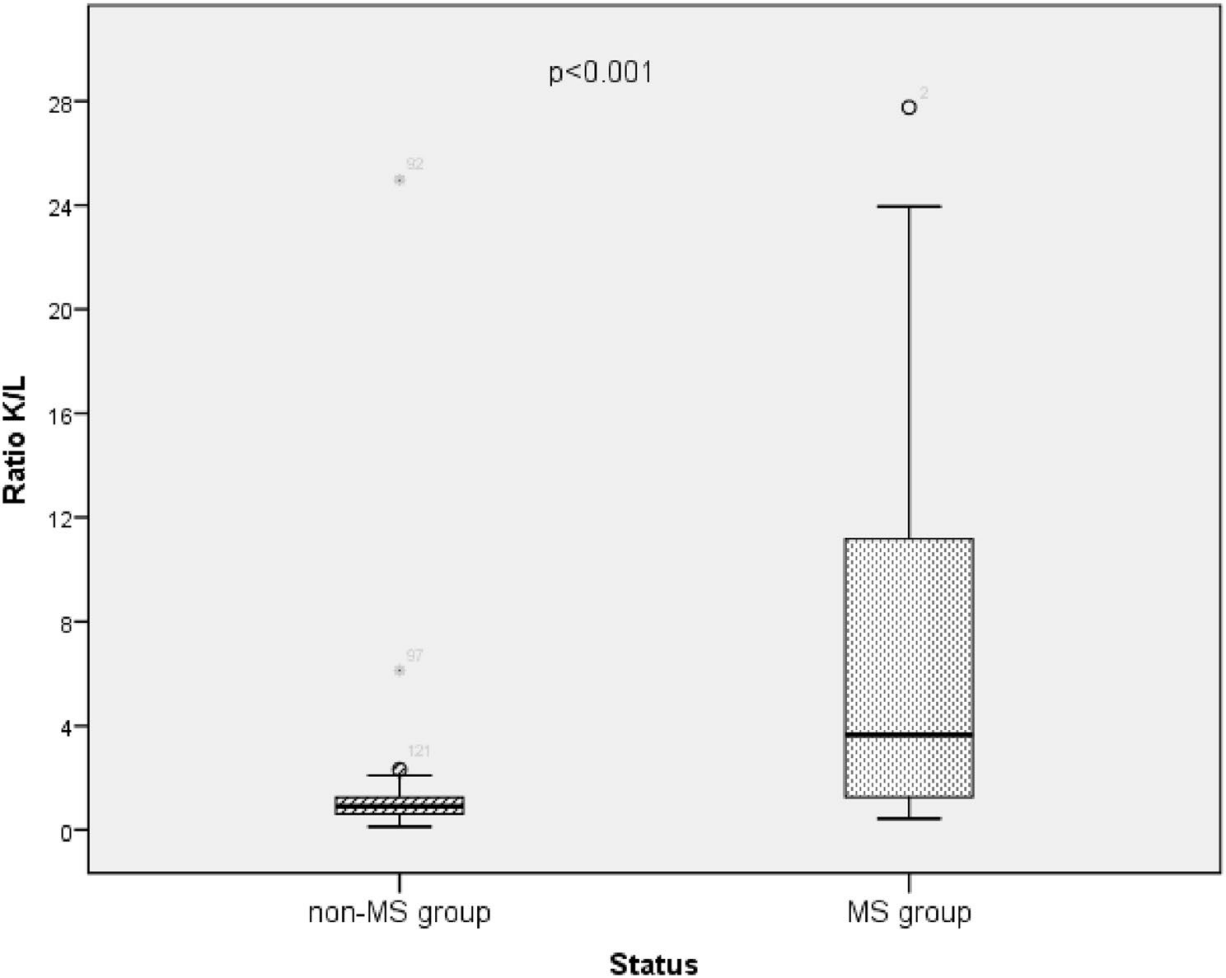
Box plots illustrating the comparative distribution of CSF κ/λ ratio in multiple sclerosis (MS) and non-MS group (U test of Mann–Whitney). Horizontal solid lines indicate medians.

The calculated mean value of the κ/λ ratio in CSF was significantly higher in the MS group than in the non-MS group (7.8 vs 3.8; p = 0.003) and in OCB-positive than in OCB-negative patients (9 vs 1.3; *p *< 0.001) (Table 2). The distribution of this parameter in the different studied groups is represented in Fig. 1. The correlation study of the different CSF levels with EDSS in MS patients demonstrated a trend to significant correlation between this score of clinical disability and the κ/λ ratio (r = − 0.3; *p* = 0.052).

Regarding serum results, levels of the 2 FLC were slightly higher in the non-MS group and OCB-negative patients.

#### Comparison of FLC metrics

##### MS vs non-MS patients

As expected, the mean value of κ index, κIgG index, and κFLC IF in MS patients were significantly higher in MS patients than in non-MS group (*p* < 0.001). The λ FLC-related parameters (λFLC index and λIgG index) were also higher in the MS group, with a significant difference for the λFLC index (Table 2).

**Figure 2:**
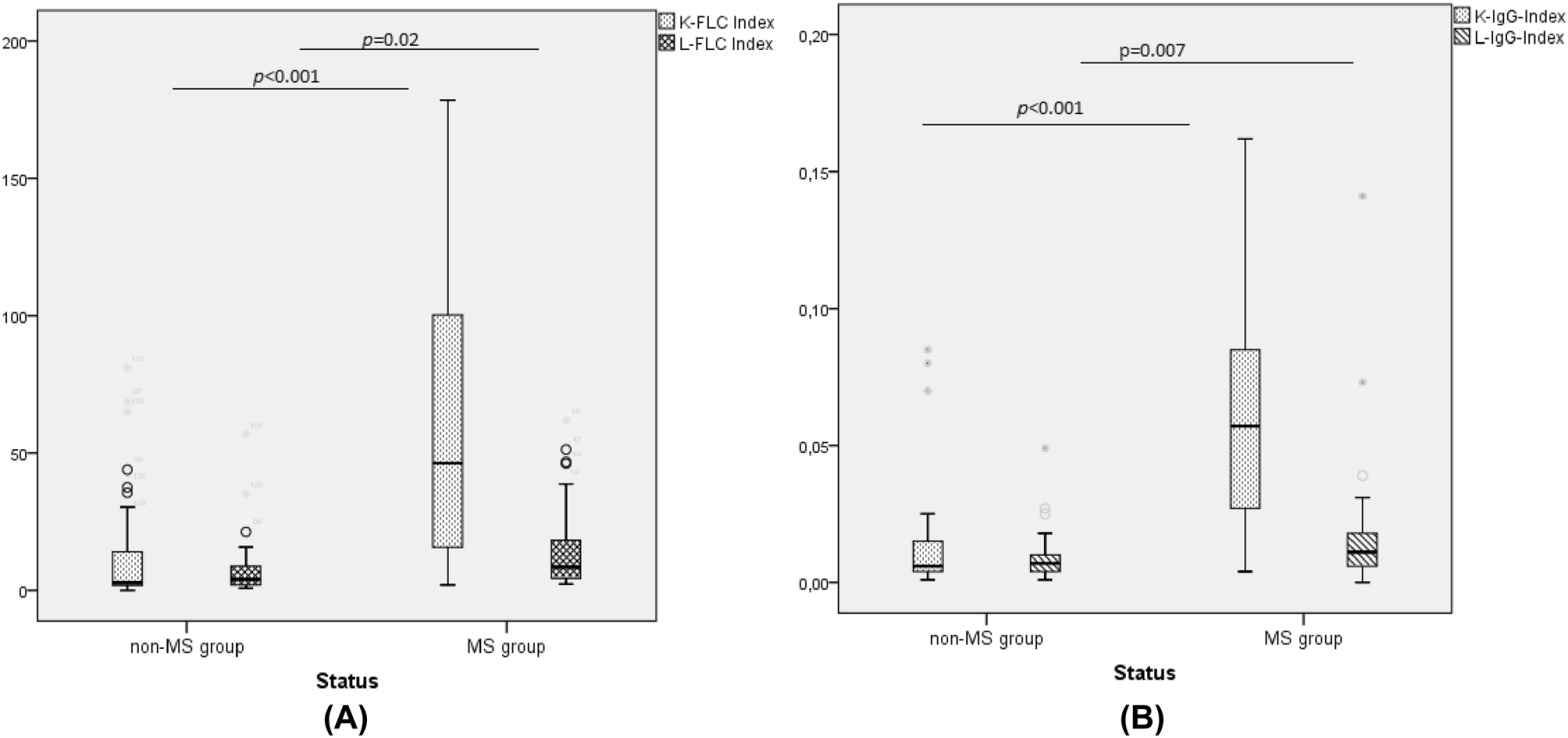
(**A**) Box plots showing the comparative distribution of κFLC index and λ FLC index in Multiple sclerosis (MS) and non-MS groups. (**B**) Comparative distribution (Box plots) of κ-IgG index and λ-IgG index in multiple sclerosis (MS) and non-MS groups (U test of Mann–Whitney). Horizontal solid lines indicate medians.

The comparison of the 4 indexes distribution between the MS and non-MS groups was illustrated in Fig. 2A,B. The quantitative analysis demonstrated that, in addition to κFLC-related indexes, λ FLC-related indexes are significantly higher in the group of patients with MS disease in comparison with the control group.

##### OCB-positive vs OCB-negative patients

Considering the total cohort, κ index and κFLC IF were significantly higher in the group where OCB was detected in comparison with the group without OCB in CSF. However, for the λ index, there was no significant difference between the compared groups. The κIgG index (and not the λIgG index) was significantly higher in presence of OCB.

**Figure 3:**
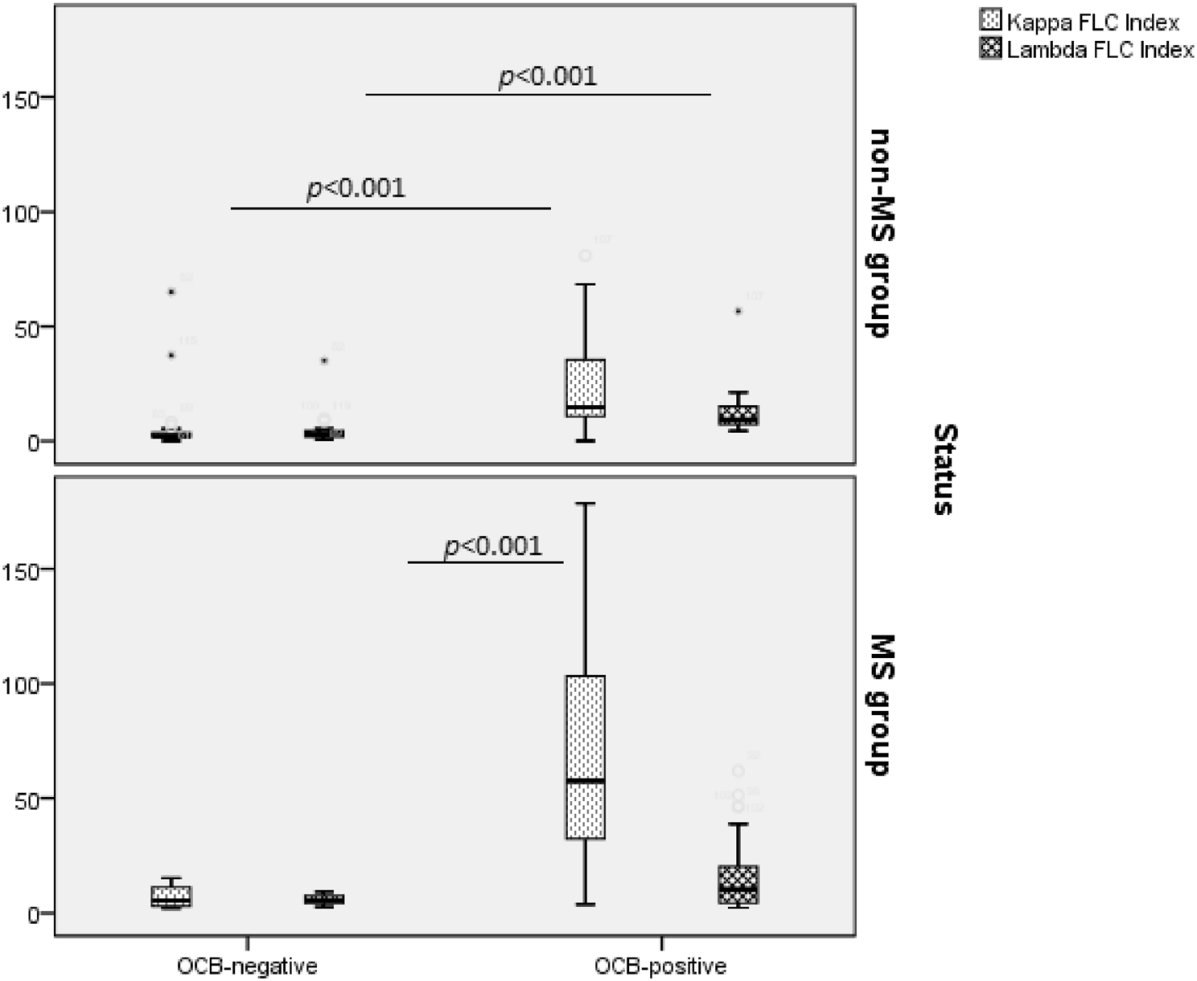
Comparative distribution of κFLC index and λ FLC index in multiple sclerosis (MS) and non-MS groups according to the oligoclonal bands (OCB) profile (U test of Mann–Whitney). Horizontal solid lines indicate medians.

The comparative distribution of the κ index and λ index according to the status (MS, non-MS) and the OCB detection (positive, negative) is shown in Fig. 3.

The mean level of κ index in OCB-negative MS patients was 16.6 ± 22.1 (1.75–88.2). This mean was 25.92 ± 29.8 in OCB-positive non-MS patients. The λFLC index and the ratio were also higher in the non-MS group with OCB. However, κIgG index and λIgG index were lower in OCB-positive non-MS patients when compared to the OCB-negative MS group.

#### FLC indexes thresholds for MS diagnosis (ROC)

**Figure 4:**
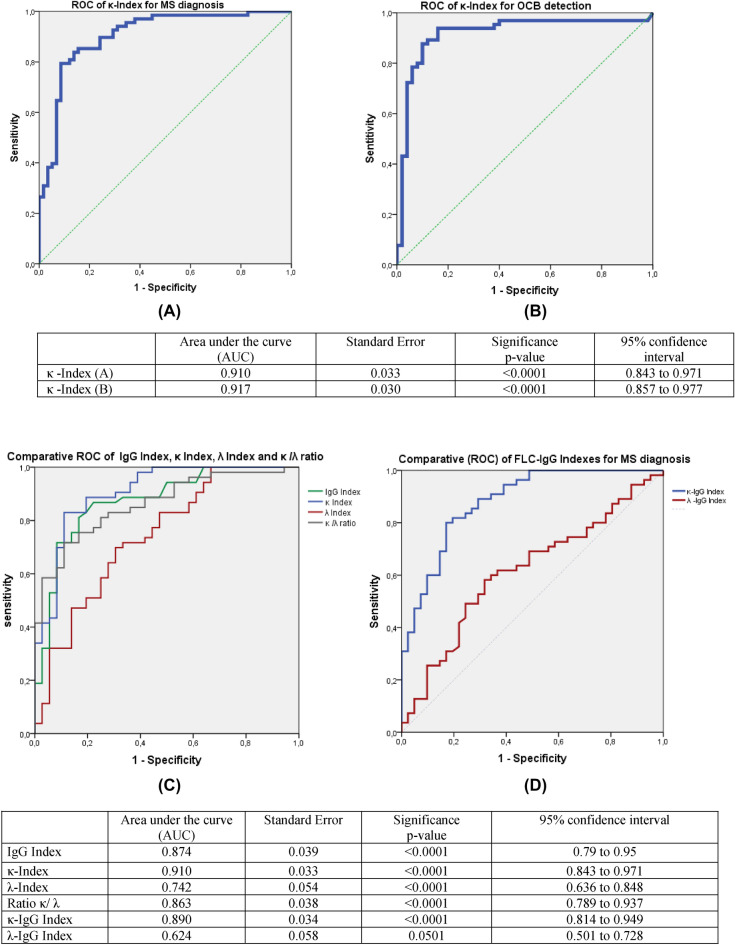
(**A**) ROC curve for the κ-index performance to discriminate MS patients from non-MS patients. (**B**) ROC curve for the κ-index performance to discriminate OCB-positive patients from OCB-negative patients. (**C**) Comparative ROC curves for IgG index, κ-index, λ-index, and κ/λ ratio to discriminate MS patients from non-MS patients. (**D**) Comparative ROC curves for κ-IgG index and λ-IgG index to discriminate MS patients from non-MS patients. *ROC* receiver operating characteristic, *MS* multiple sclerosis, *OCB*, oligoclonal band.

A ROC curve analysis was created with data from both MS and non-MS diagnosed patients. A very high performance of the κ-index for the discrimination of MS diagnosis from non-MS disorders was reflected by an area under the curve (AUC) of 0.910 (Fig. 4A). We also generated a ROC curve of κ-index for the detection of OCB in CSF in the whole sample (Fig. 4B) and found a slightly higher performance of κ-index in the discrimination of OCB-positive patients (AUC: 0.917). Thus, in our study, this parameter seems to have a very interesting capacity for discrimination in both situations. On the other hand, the comparative ROC analysis (Fig. 4C) including IgG index, κ-index, λ-index, and κ/λ ratio to discriminate MS patients from non-MS patients revealed that AUC of κ-index and κ-IgG index were slightly higher than the conventional IgG index. The other studied parameters (λ indexes and FLC ratio) were less accurate than IgG index for MS diagnosis.

According to the ROC curves relative to the discrimination of MS diagnosis (Fig. 4A,C,D), we determined the cut-off corresponding to the best performance of the parameter (combination of sensitivity and specificity) for a diagnostic purpose (Table 3). According to Table 3, it seems that using a cut-off of 14.9, κ-index has the best performance for MS diagnosis in our Tunisian population. It is notable that in our MS group, using this cut-off, there were 6 cases of OCB-positive patients with a high κ index (> 14.9) and normal IgG index. No MS patients had a profile with a normal κ index/high IgG index.

**Table 3:** Sensitivity, specificity, positive, and negative predictive values of the different studied CSF FLC-related parameters for the diagnosis of multiple sclerosis.

Cut-off	Sensitivity (%)	Specificity (%)	Youden index	PPV (%)	NPV (%)
**κ-index**					
3	98.6	60	0.586	74.4	97
3.6	97	63	0.6	75.9	94.6
6.6	91.2	73.3	0.645	80	87.2
11	87	84	0.71	86.8	83.9
14.9	83	91.7	0.747	91.8	81
20	77	93	0.7	92.9	76.5
**κ-IgG-index**					
1.07	95.4	67	0.624	76.8	93
1.6	87	79.6	0.666	82.6	84
2.4	82	86.4	0.684	86.6	81
4.8	60	94	0.54	92.8	67.5
**λ-index**					
4.9	72	60.8	0.33	70	62.3
6.7	61.1	70.2	0.32	72	60
**λ-IgG index**					
0.8	63.1	65.3	0.284	67.9	60.3

*PPV* positive predictive value, *NPV* negative predictive value.

#### κFLC reibergram

**Figure 5:**
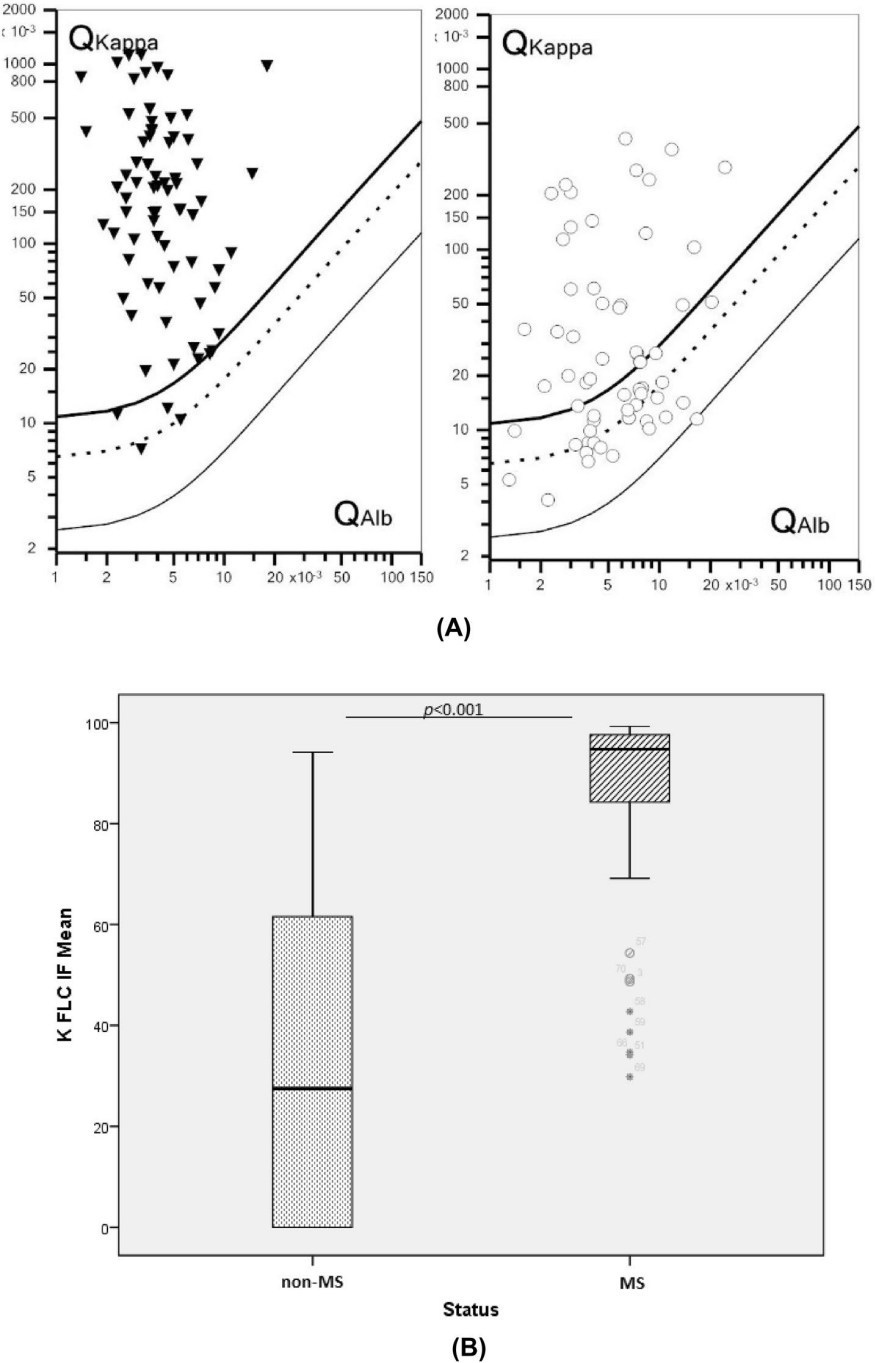
Comparative presentations of κ-FLC-related parameters in multiple sclerosis (MS) patients and controls (non-MS patients) according to the software “FLC-κ statistics and graphic program”: (**A**) κ-FLC Reibergrams showing the relation between the CSF/serum κ-FLC quotient (Q kappa) and the CSF/serum albumin quotient (Qalb) for each patient. An intrathecal synthesis of κ-FLC is revealed by a Q kappa value above the upper line, depicting the hyperbolic border line Q kappa (lim). The Q kappa mean is represented by the dashed line and the lower limit of the reference range (Q kappa low) by the lower line. (**B**) Comparative distribution of κFLC intrathecal fraction (IF) in MS and non-MS groups using U test of Mann–Whitney. Horizontal solid lines indicate medians.

**Figure 6:**
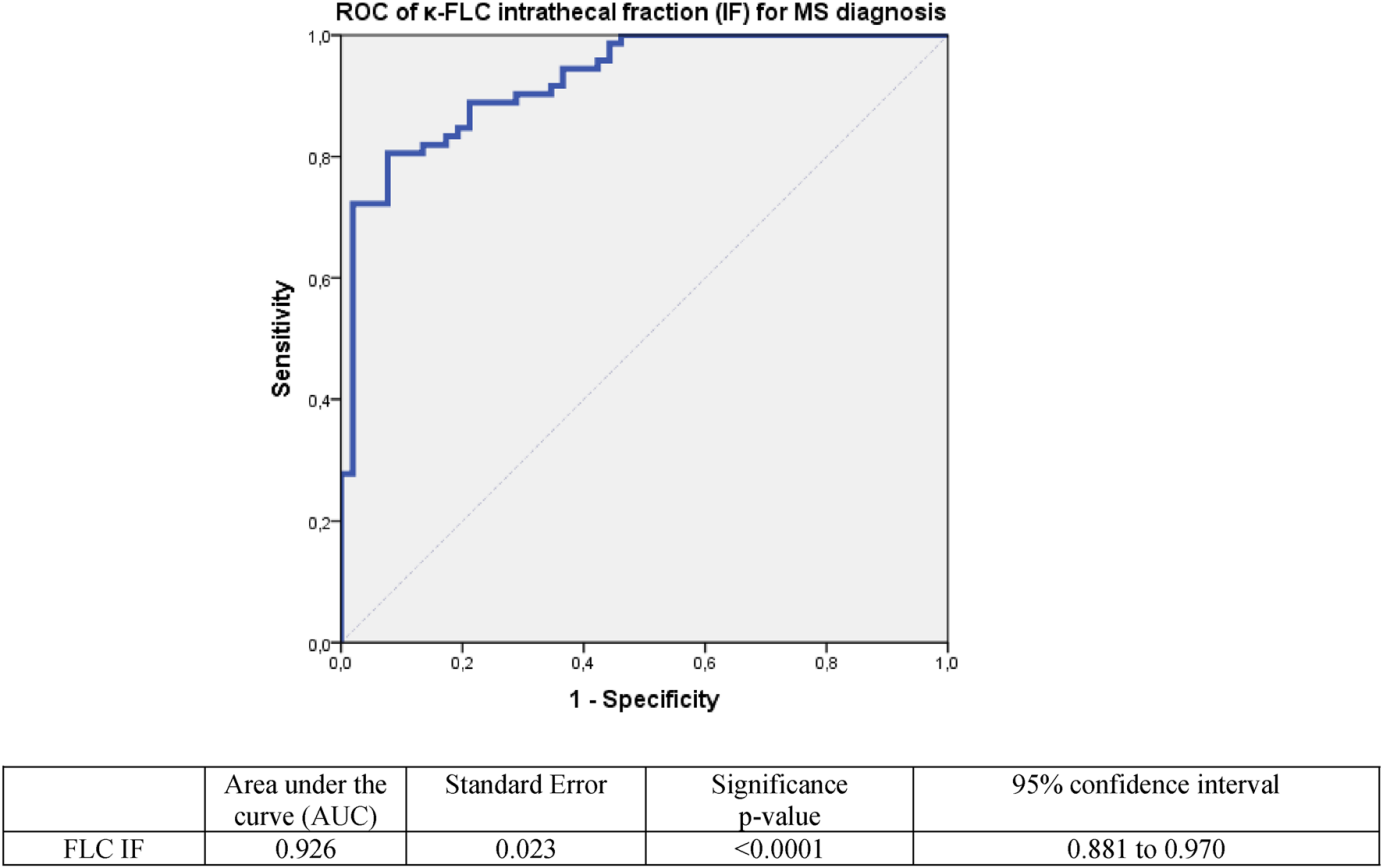
ROC curve for the κFLC IF performance to discriminate multiple sclerosis (MS) from non-MS patients. *ROC* receiver operating characteristic, *FLC IF* free light chain intrathecal fraction.

Using the software “FLC-κ statistics and graphic program” for Free Light Chains κ, we generated a graphical result for MS and non-MS patients (Fig. 5A) and we calculated and compared the mean values and the distribution of the fraction κFLC IF between groups (Fig. 5B). We also tested the performance of κFLC IF for MS diagnosis using a ROC curve (Fig. 6).

According to the ROC curve, diagnostic accuracy (AUC: 0.926) of κ-FLC IF was slightly higher than the κFLC index. For a cut-off of 54.9%, the sensitivity was as high as 92.3%, with a specificity of 75%. With a higher cut-off of 80%, the specificity reached 90.7%, with a sensitivity of 8.5%.

##### Tests combination

In OCB-negative MS patients, 5/19 (26.3%) cases had a combination of high κ index (cut-off: 14.9) and high IgG index (cut-off: 0.7). This profile (double increased indexes) was not found in any OCB-negative non-MS patients (n = 51).

### CXCL13 in CSF

In the second step of the study, the CXCL13 level was measured in the CSF of the study population (n = 160). To evaluate the comparative distribution in the presence and absence of MS diagnosis and an IS of IgG, Means, medians, and quartiles of CXCL13 levels in CSF have been calculated. The same comparison has been performed between different subgroups of MS patients (Table 4).

**Table 4:** Quantitative analysis of CXCL13 level in CSF in the study population.

	CXCL13 level in CSF [mean–median (1st Quartile–3rd Quartile)] (pg/mL)		*p-*value*
**Study population**			
Disease status	MS group (n = 82)	Non-MS (n = 78)	**0.029**
	36.6–15.3 (6.6–50.1)	27.7–7.7 (4–29.9)	
IgG index	High (> 0.7)	Low (< 0.7)	** < 0.001**
	45.3–23.8 (11.4–56)	19.1–7 (4–17.4)	
OCB profile in CSF	OCB-positive	OCB-negative	** < 0.001**
	43.2–23.5 (11.1–58.2)	19.5–6.8 (4–17.2)	
Intrathecal synthesis of IgG (Reiber)	Positive	Negative	**0.001**
	43–19 (8.1–50.4)	22.4–6.1 (4–17.6)	
**MS subgroups**			
IgG index	High (> 0.7)	Low (< 0.7)	**0.04**
	49–23.7 (9.2–59.9)	21.4–9.4 (5.1–17.2)	
OCB profile in CSF	OCB-Positive MS	OCB-Negative MS	0.22
	48.4–19.8 (9.3–59.4)	26.7–6.5 (4–18.5)	
Intrathecal synthesis of IgG (Reiber)	Positive	Negative	0.15
	42.5–18 (9.1–50.5)	33.5–7.5 (4–18.1)	

*CSF* cerebrospinal fluid, *MS* multiple sclerosis disease, *Ig* Immunoglobulin, *OCB* oligoclonal bands.

*U test of Mann–Whitney.

Significant *p* values are in bold.

**Figure 7:**
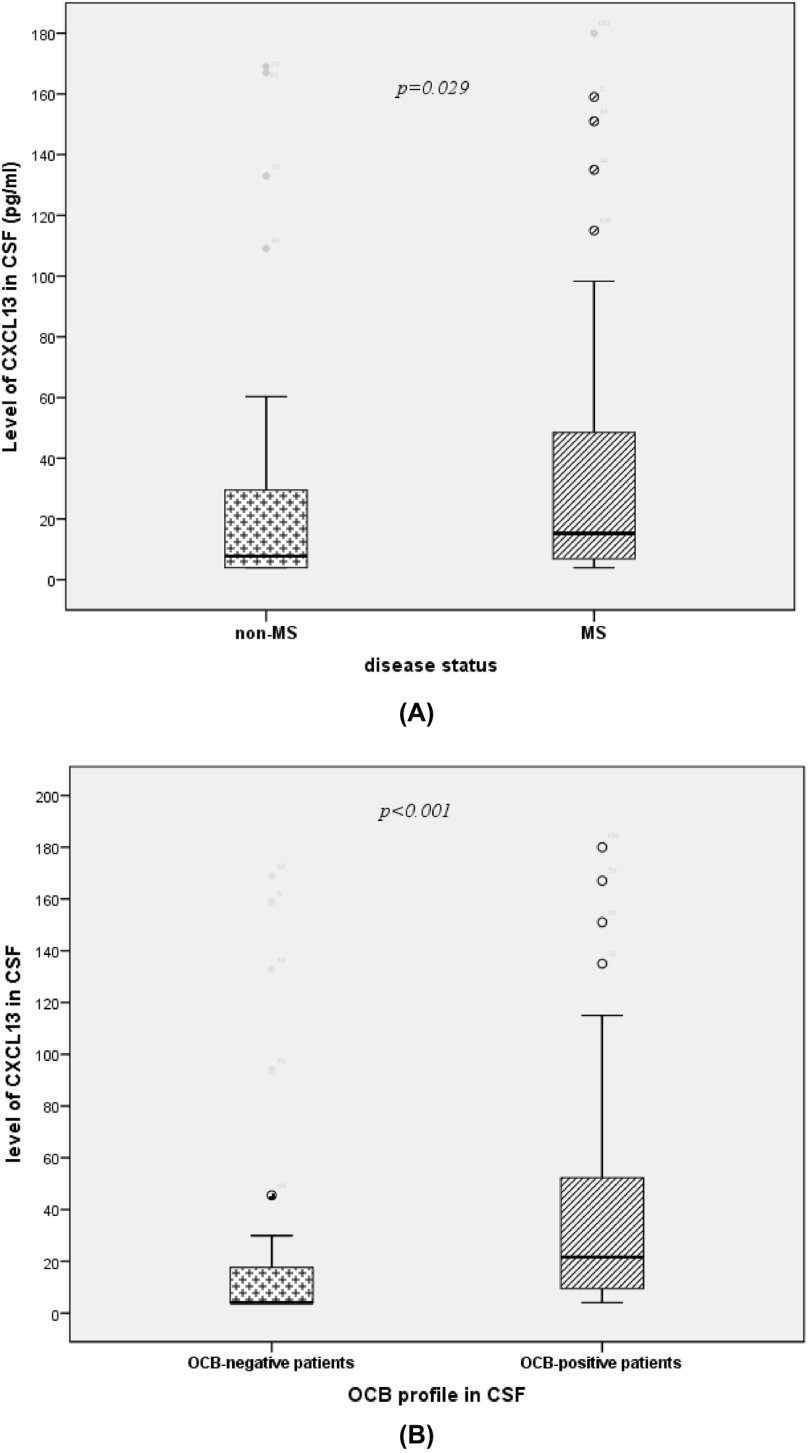
Comparative distribution of CXCL13 level in CSF: of multiple sclerosis (MS) vs non-MS patients (**A**); oligoclonal band (OCB)-positive vs OCB-negative patients (**B**) using U test of Mann–Whitney. Horizontal solid lines indicate medians.

As expected, levels of CXCL13 were significantly higher in the MS patients in comparison with the non-MS group (*p* = 0.029) (Table 4, Fig. 7A).

Considering the total population, the level of this chemokine was significantly higher in patients with a detected intrathecal humoral immune response (calculated IgG index ≥ 0.7, OCB in CSF (Isofocusing test) or an IS of IgG (Reiber diagram)) than the corresponding negative group (Table 4). The comparative distribution of CXCL13 between OCB-positive and OCB-negative patients is shown in Fig. 7B.

In the MS group, under clustering according to the previous parameters (calculated IgG index < or ≥ 0.7, positive or negative OCB in CSF, and detection or not of an IS of IgG (Reiber diagram)), the statistical significance was found only between subgroups with high and low mean of IgG index. On the other hand, the CXCL13 level in CSF was not correlated with the clinical disability evaluated by the score EDSS in the MS group.

### Correlation study of B cell-related parameters in CSF

Results of the correlation analysis of the studied B cell-related parameters (IgG, FLC κ and λ metrics and CXCL13) were summarized in Table 5. Our findings revealed that the κ index was in positive correlation with all the other studied CSF parameters. Overall, it seems that most of the FLC-related parameters (except the λIgG index) were positively correlated with the IgG index and with the level of CXCL13 chemokine.

**Table 5:** Spearman’s correlations between the different CSF variables related to the humoral immune response in the total study population (n = 160).

	IgG index	κ index	λ index	κ/λ ratio	κIgG index	λIgG index	CXCL13
κ index	r = 0.85 *p* < 0.001						
λ index	r = 0.57 *p* < 0.001	r = 0.52 *p* < 0.001					
κ/λ ratio	r = 0.72 *p* < 0.001	r = 0.84 *p* < 0.001	r = 0.041 *p* = 0.68				
κIgG index	r = 0.027 *p* = 0.002	r = 0.97 *p* < 0.001	r = 0.41 *p* < 0.001	r = 0.85 *p *< 0.001			
λIgG index	r = 0.13 *p* = 0.17	r = 0.2 *p* = 0.037	r = 0.85 *p* < 0.001	r = − 0.17 *p* = 0.08	r = 0.18 *p* = 0.064		
CXCL13	r = 0.5 *p* < 0.001	r = 0.42 *p* < 0.001	r = 0.2 *p* = 0.05	r = 0.23 *p* = 0.022	r = 0.34 *p *< 0.001	r = 0.004 *p* = 0.97	

## Discussion

Currently, B cells are reported to be major contributors to the pathogenesis of MS, an inflammatory neurodegenerative disorder of the CNS characterized by an intrathecal humoral immune response^4^. To reveal these IS of affinity-maturated IgG antibodies considered a hallmark of the disease, MS biological investigation is mainly based on the detection of IgG OCB by parallel CSF and serum samples testing using an isoelectric focusing technique, known as the gold standard for CSF assessment in this context^32^. Recently, OCB detection has been included in the revised criteria of McDonald 2017 for MS, which has permitted the substitution of the dissemination in time criterion.

However, some limitations of OCBs detection are still reported in clinical practice. From the laboratory's point of view, the imprecise number of bands in the CSF defining positive results^33^, the need for some expertise to perform the technique—a relatively laborious method, partially automatized—and the lack of standardization^34^ are some reported issues for the practice of the test and the validation of its results. In addition, OCBs detection is not specific enough for MS diagnosis^35^ and can be missed in a subgroup of authentic MS^36^. Taking all of this into account, an additional indicator may be needed to improve the biological diagnosis of MS. Recently, standard assays to measure other B cell-related biomarkers in CSF—the κ and λ FLC and the chemokine CXCL13 responsible for B cell recruitment within the CNS—have been studied and seem to improve the detection of the intrathecal B cells activity. In general, the detection of high levels of the polyclonal form of FLC was observed in many inflammatory and auto-immune disorders, which has led to testing their relevance as a biomarker of disease activity and as an attractive therapeutic target. Polyclonal FLC overproduction can occur when there is an excess production of Ig, as a consequence of chronic immune stimulation. During the inflammatory process, FLC seem to inhibit the apoptosis of inflammatory immune cells such as neutrophils and bind to a broad range of cell membranes (high affinity with monocytes).

Therefore, in this study, we aimed to optimize the diagnostic algorithm using routinely available and recently reported Laboratory tests to reveal the IS of IgG and to discriminate MS patients from non-MS patients with symptoms of central neurological disorders. Indeed, in our laboratory, we have started performing FLC and CXCL13 measurements within the routine CSF investigation for central neuroinflammation since January 2018. In the present study, we tried to compare the performance and the usefulness of different CSF parameters related to B cell activity (IgG, OCB, κ and λ FLC metrics, CXCL13) for the detection of IS and MS diagnosis, in a cohort of Tunisian patients. We also tested the κFLC data interpretation using the new hyperbolic reference range in quotient diagrams^37^. To the best of our knowledge, this is the first study interested in the diagnostic value of the recently described B cell-related parameters (Immunoglobulin FLC in CSF and their related parameters, and CXCL13 chemokine responsible for B cell recruitment within the CNS) in Tunisian and North African population. Such findings should be very helpful for MS clinicians treating diverse patient populations.

As expected, our main findings revealed that the κFLC-related parameters, in particular the κFLC index and the intrathecal fraction κFLCIF, which consider serum FLC levels as well as BBB function, reached the highest accuracy in MS diagnosis and IS detection. Regarding CXCL13 measurement in CSF, it seems to be an effective parameter reflecting the inflammatory intrathecal B cell response and may be suggested to take part in the diagnostic but mainly the prognostic investigation in MS disease.

### CSF parameters

When considering our global results of FLC measurement in CSF, 26/160 patients had an undetectable level of κFLC with our nephelometric technique, only 4 of them were diagnosed with MS and they were all OCB-negative MS patients. These findings are in line with those reported in the study of Sanz Diaz et al*.*^29^; where authors found that 160/252 (63.8%) patients had undetectable κ-FLC levels in CSF (turbidimetric technique), of whom 157 did not have MS. These results lead them to conclude that it is justified to obviate OCB testing in patients with suspicion of MS having undetectable κ-FLC levels in CSF unless there is strong clinical suspicion. Our findings comfort this proposed strategy, which should allow saving reagent costs and labor time for unnecessary OCB testing.

When comparing our CSF findings relative to each type of FLC, and as expected, κ production in CSF appears to be a much more interesting quantitative parameter than λFLC; Indeed, κFLC is associated with the IS of IgG (OCB) and with MS disease, while λ level in CSF seems to be only related to the intrathecal activity of B Lymphocytes and not specifically to the disease. Indeed, several studies from different non-African continents have indicated that during MS, abnormal intrathecal production of these FLC occur, resulting in increased κ and, to a lesser extent, λ FLC levels in the CSF of patients^24,38–48^. It has been described that after B cell activation, plasma cells secrete an excess of κ and λ FLC, in addition to intact Ig production. FLC do not appear to derive from the degradation of the whole IgG^49^. These parameters may therefore represent a potential quantitative tool to detect the IS of IgG and support MS diagnosis.

Another interesting result in our CSF findings is that the mean value of the κ/λ ratio in CSF was significantly higher in the MS group than in the non-MS group and in OCB-positive than in OCB-negative patients. In addition, our study revealed a trend for a significant correlation between the score of clinical disability (EDSS) and this ratio. In fact, it has been demonstrated that CSF κ/λ ratios, determined at the time of diagnostic lumbar puncture, may predict the progression of MS disease (EDSS at 5 years postdiagnosis) and thus may be useful as a prognostic biomarker for early therapeutic stratification^50^. Interestingly, the same study demonstrated that the high κ/λFLC ratio in CSF (good prediction of disability accumulation) seems to result from a selective expansion of κ light chain-expressing plasmablasts revealed within the CSF of MS patients and is not an epiphenomenon of FLC measurement. Other authors reported that the CSF FLC ratio may predict the conversion from CIS (clinically isolated syndrome) to MS disease^38^.

### FLC metrics

Overall, κFLC metrics were more performant for MS diagnosis and detection of the IS than λFLC-related parameters. Results of the comparative sensitivity, specificity, PPV, and NPV of the κ and λFLC metrics for MS diagnosis in our Tunisian population confirmed the reported vast superiority of κFLC-related parameters described in other populations. In particular, the κFLC index and κFLC IF showed the best accuracy for MS diagnosis. The higher clinical relevance of κFLC in MS could be relatively explained by the fact that the κ chain is rearranged first during Ig production and is consequently quantitatively more dominant in the human body. In addition, λFLC are usually found in a dimeric form while κFLC are mostly monomeric^51^.

On the other hand, FLC, as proteins detected in the CSF, originated in part from the blood by diffusion across the blood–brain barrier (BBB) but could also be produced within the CNS during pathological conditions, such as MS disease^52^. It is therefore necessary to differentiate the locally synthesized intrathecal fraction of FLC from the blood-derived fraction. Two approaches to studying the intrathecal fraction of κFLC have been established and evaluated: the known linear approach (in analogy to IgG index) by calculating the FLC index (for both types of FLC) with reference to a determined cut-off, and a non-linear function for the κ chain (in analogy to Reiber’s formula) by coupling each QFLC (CSF FLC/serum FLC) to its corresponding Qalb dependent upper normal limit (Qlim FLC). In the Two approaches, Qalbis considered a well-established parameter for BBB status and function, and the inter-individual variability of serum FLC levels is taken into account^52^. Non-linear functions are in general considered more representative of the physiological conditions and appear to be superior in case of barrier dysfunction^53,54^. However, the linear IgG index formula is relatively simpler to perform in routine.

In our study, and according to the ROC curves analysis, κ metrics were overall more accurate than the classical Total IgG index for MS diagnosis. In particular, κFLC IF (AUC: 0.926) accuracy was slightly higher than the κFLC index (AUC: 0.910) and than IgG index (AUC:0.874). This finding is in line with the results of previous studies where κFLC IF has been found to be the more accurate metric compared to κ index and conventional parameters^55^. Evaluation of κFLC with this hyperbolic reference range in quotient diagrams seems to decrease the number of false-positive/-negative cases in the presence of low/high albumin quotients, which allows a most reliable interpretation of the data^56^. Other authors reported that both linear and non-linear approaches have a similar performance in patients with normal or only slightly elevated Qalb^57^ and that diagnostic accuracy does not differ between the κFLC index and κFLCIF^58–60^. Therefore, we can consider κFLC IF as a very promising parameter in the context of MS suspicion, having at least the same diagnostic accuracy as the κ index.

Another interesting finding in our study is the high percentage of the intrathecal fraction IF detected using the diagram in our MS patients (mean 86.87%). Indeed, it has been described that this intrathecal fraction of CSF FLC is greater than 80% in most MS patients^61^, which can reflect that the contribution of blood-derived FLC to total CSF FLC concentration is very low in MS cases with intrathecal synthesis. This observation has led some authors to use QFLC (without considering BBB function)^59,62–64^, or even the absolute CSF FLC concentrations^65^ for MS laboratory investigation.

With a cut-off of 14.9, the κFLC index showed a sensitivity of 83%, a specificity of 91.7%, and a positive predictive value (PPV) of 91.8%. These values are within the correspondent range of values reported for this parameter in different populations^25,39–41,66,67^. Interestingly, both sensitivity and specificity of the κFLC index were higher than OCB detection (using the Isofocusing test) for MS diagnosis in our same Tunisian population (77.6% and 81.8%, respectively as mentioned in our previous published study)^68^. By focusing on our MS group, there were 6 cases of OCB-positive patients with a high κ index (> 14.9)/normal IgG index and no MS cases with the inverse profile. This could reflect, in the context of MS suspicion, the higher sensitivity and the more reliability of the κ index as a quantitative test for the detection of intrathecal antibody production. In the OCB-negative MS subgroup, 5/19 (26.3%) cases had a combination of high κ (cut-off: 14.9) index and IgG index. No OCB-negative non-MS patients (n = 51) showed this profile, which could reveal the usefulness of the combination of κ and IgG indexes in case of strong MS suspicion with a negative result in the OCB detection test.

Regarding MRZ Reaction testing in our population, it was found as a very specific test (91.9%) with a very good PPV (93.9%) but a relatively moderate sensitivity (65%) in another previous study performed in our laboratory^69^. MRZ reaction has been proposed as a very specific-second line test for CSF investigation, especially when OCB is negative or equivocal and/or when the clinical context is confusing. In the present comparative study, the κFLC index had similar specificity to the MRZ reaction with much better sensitivity.

As mentioned above, the intrathecal fraction κ-FLC IF determined using the software “FLC-κ statistics and graphic program” for κFLC showed a slightly higher diagnostic accuracy than the κFLC index. For a cut-off of 54.9%, the sensitivity reached 92.3% (specificity: 75%). With a higher cut-off of 80%, the specificity was 90.7% (sensitivity: 80.6%).

In addition to κFLC index and κFLC IF, the concentrations of FLC and IgG have been evaluated in our study using the same formula for the diagnosis of MS (κIgG index and λIgG index), as an innovative approach which has been just described and tested^70^. CSF concentrations of FLC and IgG are known to be increased in MS patients, but they have never been used in the same algorithm. Our findings showed that the κIgG index (and not the λIgG index) has a diagnostic accuracy (AUC: 0.890) close to that of the κFLC index (AUC: 0.910) and is significantly higher in patients groups with MS and with positive OCB in CSF. This new tested parameter also showed interesting sensitivity and specificity for MS diagnosis when considering a cut-off of 2.4. In the study of Gudowska-Sawczuk et al.^70^, authors reported a significant difference in κIgG index and λIgG index values between MS patients and controls having other neurological disorders, with a higher diagnostic significance for κIgG index compared with the λIgG index in this context. Regarding OCB, in the same study, κIgG appeared to be useful in pattern differentiation. This parameter was significantly higher in patients with type 2 patterns in comparison with those with patterns type 1 and 4. Only the values of this parameter were markedly higher in patients with pattern type 3 than patterns type 4 and 1. Taking into account all these data, this parameter could be considered a good candidate for further studies with a larger patient group size.

### CXCL13 level in CSF

In the last part of our study, we analyzed another B cell-related parameter in CSF, which is the molecule CXCL13, also called *B-Cell Attracting chemokine 1 (BCA-1) or B-Lymphocyte chemoattractant (BLC).* This chemokine plays an important role in B cell recruitment within the CNS during inflammatory disorders. It has a crucial role in the development of secondary lymphoid nodules and the regulation of lymphocyte migration. In these conditions, CXCL13 is mainly expressed by monocytes/macrophages/microglia but can also be produced by follicular dendritic cells. The Receptor for this molecule (CXCR5/BLR1) is found mainly on B cells and T-cells (about 20% of CSF TCD4+) and monocytes- macrophages^26,71,72^.

In our study, the CXCL13 level in CSF was significantly higher in the MS group in comparison with the non-MS group (mostly inflammatory conditions of CNS). In fact, several studies reported CXCL13 as a potential marker of inflammation in CSF and high levels of this molecule have been reported in CSF during MS (RRMS, PPMS, and CIS disease courses)^26–28^ and NMO conditions^73^, while very high CSF levels have been described in MS mimics, such as CNS lymphoma, neuroborreliosis (where CXCL13 is described as a specific biomarker) and viral miningitis^74^. The CSF levels of CXCL13 in our MS group seem slightly higher than in other studies^26–28,75,76^. Indeed, in our study, most of the patients had a RRMS and all of them were in relapse at the time of sampling. It has been reported that during MS relapse, Levels of CXCL13 are higher and the positive correlations between these levels and other molecules that are essential for B-cell recruitment, survival, and maturation (B-cell activating factor (BAFF), IL-6, and IL-10) seem to be crucial to driving the B-cell response^26,28,75,76^. Regarding levels in the non-MS group, there is a big heterogeneity of findings since it depends on the type of other inflammatory or non-inflammatory diseases included, where production of CXCL13 in the CNS could vary greatly^27,73,76^.

On the other hand, considering the total population of our study, CXCL13 was significantly higher in patients with a positive intrathecal humoral immune response (IgG index ≥ 0.7, OCB in CSF (the Isofocusing test) or an IS of IgG according to the Reiber diagram)) than the corresponding negative group. In addition, CSF levels of this chemokine were in positive correlation with CSF markers of intrathecal B cell activity, in particular, IgG index, κFLC metrics (κ index; κIgG index), and κ/λ ratio. These findings were consistent with the results of other studies where CXCL13 levels were demonstrated to correlate strongly with the IS of Ig (IgG level in CSF, IgG index, number of OCB, BAFF level in CSF) as well as the presence of B cells, plasmablasts and T cells (or the number of inflammatory cells)^28,72,76,77^.

Some authors also mentioned that in the MS group, patients with IS of IgG had higher CXCL13 levels than patients without IgG synthesis, which was observed in our study with a significant difference. Therefore, CXCL13 seems to be a marker of intrathecal inflammation rather than a specific parameter for MS diagnosis. It is an important mediator in the inflammatory cascade associated with the polyspecific intrathecal B cell response classically revealed by IgG index or OCB. This chemokine could therefore be a potential candidate biomarker to evaluate the efficiency of B-cell targeting therapy during MS. This hypothesis has been studied in Rituximab-treated MS patients, where it has been shown that CXCL13 levels in the CSF decrease significantly under treatment^78^. In addition, as a factor that attracts and maintains B and T cells in inflamed CNS lesions, both CXCL13 and its receptor CXCR5 have been suggested as therapeutic targets in MS^72^.

Regarding MS prognosis, much evidence demonstrated that CXCL13 could be considered an effective marker of severity and activity of the disease. This chemokine has shown an association with disease exacerbations and unfavorable prognosis in RRMS. A significant positive correlation between CXCL13 level in CSF and relapse rate, clinical disability (EDSS), and the number of MRI lesions have been described. In addition, a high level of this molecule was predictive of CIS conversion to MS^76^. In our study, no correlation with EDSS results was found, which can be explained by the relatively small size of the MS group. In the study of Di Sano et al., the CXCL13 index was calculated, and this parameter was also reported to be an excellent candidate prognostic biomarker for predicting MS disease activity^79^.

Regarding patient stratification, the CXCL13 level in CSF could identify an MS subgroup with higher CSF leukocyte counts and early evidence of cortical thinning reflecting the disease severity^75^.

In clinically and radiologically stable patients, it has been reported that a significant proportion still showed increased CSF levels of CXCL13 (in addition to neurofilament) indicating residual disease activity. Thus, this chemokine seems to be more sensitive than clinical and MRI features in revealing disease activity^80^.

We propose that CSF CXCL13 measurement, as an effective parameter reflecting the inflammatory intrathecal B cell response, can be part of the diagnostic but mainly the prognostic work-up in MS. It has been particularly reported as a helpful tool in future treatment decisions.

To the best of our knowledge, this is the first study interested in the diagnostic value of FLC in CSF and their related parameters in the Tunisian and North African populations. In addition, our study has interestingly tested concomitantly almost all the κ and λ FLC metrics described so far (CSF levels of κ and λ FLC, κ/λ CSF ratio, κ, and λ FLC indexes, κ, and λIgG indexes, κFLC IF), in addition to the recently reported software “FLC-κ statistics and graphic program”. The performance of these parameters has been studied comparatively, and they have also been compared to the performance of routine tests (especially OCBs and MRZ reaction) reported in our previous studies including the same populations. Furthermore, our study is one of the very rare publications interested in the concomitant evaluation of CXCL13 (responsible for B cell recruitment within the CNS) and FLC levels in CSF during MS. There is a single study^81^ where both biomarkers have been evaluated in CSF during rituximab treatment, however, this study included only 9 treated MS patients, and the statistical analysis was very limited with no relevant results regarding these 2 parameters. However, this study presents some limitations, such as its monocentric characteristic and the relatively small number of patients included.

Finally, we aimed to optimize the algorithm of routine CSF investigation for MS diagnosis and prognosis by adding the new effective, simple, available, and standardizable laboratory parameters. Based on our previous^68,69^ and present findings and the above literature data, we suggest that:

- Initial measurement of automatized κ-FLC in CSF allows obviating OCB testing in case of undetectable levels in patients with suspicion of MS unless there is strong clinical suspicion.
- Complete Calculation of IgG index, κ-index values, and evaluation of FLC-κ Reibergram (κFLC IF) as the next automatized, relatively simple, rapid, and standardizable step of effective screening for IS detection and therefore, MS diagnosis to obtain the highest sensitivity and accuracy.
- This CSF investigation is followed by the CSF Isofocusing test (OCB detection) if available, with a combined interpretation of all previous parameters (especially the κ and IgG indexes) and the clinical context.
- As an optional additional step, in remaining confusing cases, MRZ Reaction could be performed (highest specificity for MRZ Reaction with a good sensitivity when combined with OCB detection).
- CXCL13 measurement in CSF can take part in the armamentarium for the work-up in MS, especially for prognostic evaluation (good prognostic biomarker for MS activity and prediction of treatment response).

In conclusion, this study highlights the usefulness and accuracy of CSF examination in the diagnosis of MS, by demonstrating the high performance of the newly described CSF B cell-related parameters (especially the intrathecal κFLC metrics) in this context, in addition to the well-established (OCB detection, IgG index…) routine laboratory tests.

Overall, the determination of FLC metrics in CSF seems to have many advantages over the established CSF parameters detecting the IS of IgG. They appear to be valuable and supportive additional quantitative parameters in CSF investigation for MS diagnosis but may not replace the gold standard OCB detection. These clinically reliable parameters are technically simple, rapid, standardizable, and offer objective results. In particular, κ-related metrics are more sensitive than the other quantitative and qualitative tests for IS detection with good specificity. The calculation of κ index and the interpretation of κ-FLC data using a diagram with a hyperbolic reference range (considering the BBB status) showed the best accuracy for MS diagnosis. However, FLC-related parameters do not inform about the clonality of the IS, which is one of the advantages of OCB detection using the Isofocusing test. On the other hand, CXCL13 appears as an established mediator of the inflammatory cascade associated with the polyspecific B cell response and it has been reported to be promising in prognosis evaluation and prediction (activity of the disease, treatment decisions…). A CSF testing combining conventional and new biological tools (quantitative standardizable tests) may certainly improve the performance of MS investigation.

## Data Availability

The datasets used and/or analyzed during the current study are available from the corresponding author on reasonable request.

## Abbreviations

MS: Multiple sclerosis
CIS: Clinically isolated syndrome
OCB: Oligoclonal bands
CSF: Cerebrospinal fluid
FLC: Free light chains
AUC: Area under the curve
CNS: Center nervous system
Ig: Immunoglobulin
CXCL3: C-X-C motif chemokine ligand 13
IS: Intrathecal synthesis
EDSS: Expanded disability status scale
SD: Standard deviation
NMOSD: Neuromyelitis optica spectrum disorder
PPV: Positive predictive value
IF: Intrathecal fraction
ROC: Receiver operating curve
